# Sensory Characteristics and Nutritional Quality of Food Products Made with a Biofortified and Lectin Free Common Bean (*Phaseolus vulgaris* L.) Flour

**DOI:** 10.3390/nu13124517

**Published:** 2021-12-17

**Authors:** Francesca Sparvoli, Silvia Giofré, Eleonora Cominelli, Elena Avite, Gianluca Giuberti, Diomira Luongo, Edoardo Gatti, Marta Cianciabella, Giulia Maria Daniele, Mauro Rossi, Stefano Predieri

**Affiliations:** 1 Institute of Agricultural Biology and Biotechnology, National Research Council, Via Bassini 15, 20133 Milano, Italy; giofresilvia@gmail.com (S.G.); cominelli@ibba.cnr.it (E.C.); 2 Dipartimento di Scienze Agrarie, Alimentari e Ambientali, Università Politecnica delle Marche, 60131 Ancona, Italy; 3 Blumen Group SPA, Corso Savona 168, 14100 Asti, Italy; elena.avite@blumen.it; 4 Department for Sustainable Food Process, Università Cattolica del Sacro Cuore, Via Emilia Parmense 84, 29122 Piacenza, Italy; Gianluca.Giuberti@unicatt.it; 5 Institute of Food Science, National Research Council, Via Roma 64, 83100 Avellino, Italy; diomira.luongo@isa.cnr.it (D.L.); mauro.rossi@isa.cnr.it (M.R.); 6 Institute for BioEconomy, National Research Council, Via Piero Gobetti 101, 40129 Bologna, Italy; edoardo.gatti@ibe.cnr.it (E.G.); marta.cianciabella@ibe.cnr.it (M.C.); giuliamaria.daniele@ibe.cnr.it (G.M.D.); stefano.predieri@ibe.cnr.it (S.P.)

**Keywords:** α-amylase inhibitor, biofortification, lectins, nutritional enhancement, phytic acid, predicted glycemic index, sensory analysis, temporal dominance sensation

## Abstract

Common beans (*Phaseolus vulgaris* L.) are an important source of nutrients with beneficial effects on human health. However, they contain lectins, that limit the direct use of flour in food preparations without thermal treatment, and phytic acid, that reduces mineral cation bioavailability. The objectives of this research were: to obtain biofortified snacks and a cream using an untreated common bean flour devoid of active lectins (*lec*^−^) and with reduced content of phytic acid (*lpa*) and to evaluate the sensorial appreciation for these products. The main results of the present work were: the products with the *lpa lec*^−^ flour did not retain residual hemagglutinating activity due to lectins; they showed higher residual α-amylase inhibitor activity (from 2.2 to 135 times), reduced in vitro predicted glycemic index (about 5 units reduction) and increased iron bioavailability compared to the products with wild type flour; products with common bean flour were less appreciated than the reference ones without this flour, but the presence of an intense umami taste can be a positive attribute. Results confirmed that the use of the *lpa lec*^−^ flour has important advantages in the preparation of safe and nutritionally improved products, and provide useful information to identify target consumers, such as children and elderly people.

## 1. Introduction

Legume seeds (pulses) are an excellent and inexpensive source of proteins, dietary fiber, vitamins, minerals, and bioactive components, and their consumption is associated with multiple health benefits, such as lower risk of incidence of cancers, type II diabetes, or cardiovascular diseases [1,2,3,4,5]. However, legume consumption per capita has not increased in the last three decades, despite massive population growth [6], and in several countries estimated average legume consumption does not meet recommendations. In recent years, public awareness of the nutritional benefits of pulses, as part of sustainable food production for food security and nutrition, has increased. As a result, attention to legume research has generally increased. Furthermore, innovative food alternatives that promote legume consumption and that can provide alternatives to satisfy consumer demand for healthy foods have emerged. These novel foods incorporate legumes into bakery products, pasta, soups, cereals, tortillas, meat substitutes, beverages, and snacks (recently reviewed by [7]).

Snacks occupy a considerable slice of the ready-to-eat food market and are appreciated by consumers of all ages, eaten during work-breaks by adults and during school recreation by children and adolescents. In some cases, snacks have become meal substitutes; the tendency to consume low-value snacks instead of having a complete and balanced meal is frequent and common, mainly among teenagers. This phenomenon has been associated with an increase in the body mass index and poor academic performance of students [8]. The increased consumption of energy-dense, nutrient-poor snacks is one of the major and growing concerns associated with the alarming trend of overweight, obesity and metabolic disorders worldwide [9]. This trend is also related to undesirable changes in snack-food patterns, including an increase in portion sizes, added sugars, total fats, saturated and trans-fats [10]. For this reason, the snack food market is currently searching for healthier products, characterized by enhanced nutritional value, a lower caloric content and a more balanced composition, to satisfy the changing habits of adults and children and to prevent the development of chronic diseases. Furthermore, overweight and obesity, which have always been considered a problem particularly in high-income countries, are currently dramatically increasing in low-income countries that now face concerns associated both with malnutrition and those associated with obesity [11].

Because of their high nutritional value and their positive performance in heat-treated products, legume flours are suitable ingredients for use in baked goods, and bean flour seems to receive a positive response in the formulation of ready-to-eat snacks [12,13,14,15,16]. However, legume-based snacks face challenges such as eliminating or reducing antinutritional factors (e.g., lectins, enzyme inhibitors, α-galacto-oligosaccharides, phytic acid, tannins) and meeting consumer acceptance, including texture, flavor, appearance, color, and final product approval. Sensory analysis evaluates the attributes related to perception at consumption, predicts consumer acceptance drivers, and helps identify target consumers. Sensory evaluation has been applied to bakery products containing legumes, including gluten-free [17,18] and low-fat [19] biscuits, and snacks for children [20]. Furthermore, it must be kept in mind that consumer preferences have changed over the years and nowadays food products that could provide health benefits are preferred by consumers [21].

The common bean (*Phaseolus vulgaris* L.) is an essential source of proteins and many macro and micronutrients. Therefore, its consumption has beneficial effects on human health and related pathologies, such as reducing cardiovascular diseases and diabetes mellitus, preventing different types of cancer, and controlling some metabolic functions [22]. Furthermore, because of their characteristics, beans can be introduced in gluten-free, vegetarian, and vegan diets as an optimal source of proteins. Moreover, the introduction of beans into popular industrial products, especially snacks, may be helpful to attract people and increase the use of legumes within different food formulations so that all groups of consumers can benefit from the positive effects of common beans. Moreover, they are an essential and complete staple food in many developing countries, where ‘hidden hunger’ is a critical challenge and common bean represents one of the most common crops [23].

Despite their positive characteristics, beans also contain some antinutritional compounds, such as lectins and phytic acid [24]. It is well known that consumption of raw or inadequately cooked beans causes poisoning, characterized by extreme nausea, vomiting, diarrhea, severe acute gastroenteritis and intestinal malabsorption [25,26,27]. This toxicity has been ascribed to the presence of active lectins [28], one of the major classes of common bean storage proteins. For these reasons, the presence of lectins strongly limits the direct use of common bean flour without any thermal treatment to prepare baked products, as the cooking conditions do not always ensure the complete inactivation of these toxic proteins [12]. In addition, the presence in common bean seed of high amounts of the strong cation chelator phytic acid reduces the bioavailability of important minerals such as iron, zinc, potassium, calcium, and magnesium that, at physiological pH, easily precipitate as phytate salts [29].

In recent decades, most research has sought, on the one hand, to develop approaches aimed at reducing legume seed antinutrients and improving their excellent properties and, on the other, to find possible ways to sensitize consumers to their regular consumption. For this purpose, not only well-tested technological methods (such as germination, extrusion and heat treatments) exist [13,30,31,32], but even genetic approaches can be employed [24,33]. Moreover, some technological modifications can modify some nutritional and antioxidative properties. For instance, migration of compounds with high antiradical activity to brine may significantly diminish bioavailability of active compounds because the brine is usually discarded before consumption of canned legumes [34]. Genetic approaches, by exploiting natural and induced biodiversity, were successfully used to isolate lines devoid of active lectins (*lec*^−^ [35,36]) and with low seed phytic acid content (*lpa* [37,38]). In common bean seeds, major lectins, the erythroagglutinating and leucoagglutinating phytohemagglutinins (PHA-E and PHA-L, respectively), and the α-amylase inhibitor (α-AI), also known as phaseolamin, occur at the same genetic locus, called APA [39]. α-AI can inhibit mammalian α-amylases, thus inhibiting starch digestion [40] and is widely used as an active ingredient of commercial starch blocker preparations for the control of body weight [41]. Moreover, α-AI can also reduce post-prandial glucose plasma levels, insulin C-peptide and gastric inhibitory polypeptide in healthy and diabetic subjects [42,43]. Through the screening of natural biodiversity, followed by breeding, genotypes showing differences in the presence of PHAs and α-AI have been isolated [35,36]. For example, the cv. Lady Joy, devoid of active PHAs and with an active α-AI, was obtained through the introgression into a commercial cultivar of an inactive PHA, the so-called “pinto lectin”, present in only 10% of common bean genotypes [35].

Two allelic common bean *lpa* mutants, affecting the PvMRP1 phytic acid transporter, have been isolated and characterized to date, showing a 75–90% reduction in seed phytic acid content [37,38]. From an in vivo study, iron absorption per test meal from the *lpa1* seeds was higher than from wild type beans, showing that the *lpa1* beans are biofortified [44]. However, the following study found that the *lpa1* beans could cause adverse gastrointestinal symptoms due to a hard-to-cook (HTC) phenotype concomitant with increased thermal stability of lectins in these lines [27]. As recently shown, both traits depend on the genetic backgrounds in which the *lpa1* mutation is present [45].

Unprocessed common bean flours obtained from the Lady Joy genotype, and the *lpa1* mutant have been used to prepare biscuits [12]. Results showed that if flour derives from conventional common bean, lectin activity is still detectable after baking; consequently, it has been suggested to avoid using unprocessed traditional flour of beans to produce baked products. Conversely, the unprocessed Lady Joy flour (*lec*^−^) is safe, as no lectin activity is present in the biscuits. Moreover, it was shown that baking did not fully inactivate α-AI, a trait that may contribute to lowering the predicted glycaemic index (pGI) of the biscuits. When the *lpa1* flour was used to prepare biscuits, a 50% reduction of phytic acid content in the final product was observed [12].

In a previous study [12] the use of different bean flours devoid of specific antinutrients to prepare biscuits was analyzed. Moreover, different proportions of bean flours in different composite flour formulations (e.g., with wheat and/or maize flours) were used. In the current study a wider range of bean-based products, made with an advanced bean genotype that combines the reduction of phytic acid (*lpa*) with the absence of an active lectin (*lec*^−^) while maintaining the accumulation of α-AI, were analyzed. Crackers with two different proportions of bean flour, two different types of biscuits and a cream, with a content of bean flour ranging from 9% (cream) up to 38% (Cracker 2) were produced. Our general purpose is to promote bean consumption by developing biofortified and low antinutrients beans which are well suited for directly use as bean flours. More specifically, in this study, we aimed, on the one hand, to verify how some nutritional characteristics of the bean flours (e.g., activities of the α-AI and the lectin, iron bioavailability) were influenced by different formulations and/or cooking conditions and, on the other hand, to assess the sensory properties and related acceptance level of bean-based products, according to a trained evaluation panel.

## 2. Materials and Methods

### 2.1. Plant Materials

Bean flours were obtained from a wild type (wt) *P. vulgaris* L. genotype, belonging to the bean market class Borlotto and an *lpa lec*^−^ genotype with the same genetic background of Borlotto. This genotype (*lpa lec*^−^) was a BC_1_F_5_ line derived from a cross between a BC_2_F_2_ *low phytic acid* (*lpa*) line [37] and the Lady Joy genotype [12], characterized by the absence of active lectins (*lec*^−^).

### 2.2. Products Preparation

Composite flours, containing different percentages of the bean flour, wheat flour type 2, whole wheat flour and buckwheat flour were used to prepare biscuits, crackers and a cream. Recipes were developed with the technical assistance of the backers and pastry chefs Mr. Matteo Consolo and Mr. Ferruccio Farioli at ENAIP (Ente Acli Istruzione Professionale) Lombardia, Busto Arsizio (VA, Italy). Crackers with two different amounts of bean flour, two types of biscuits, and a cream were produced. Formulations are reported in Table 1.

Crackers and biscuits were both prepared in three variants, using the flours from the wt or the *lpa lec*^−^ bean genotype or replacing the common bean flour with an equivalent amount of wheat flour type 2 or whole wheat flour (Biscuit 2). Cream was prepared with the *lpa lec*^−^ bean flour or with rice flour. Crackers and biscuits prepared with the two different common bean flours (wt and *lpa lec*^−^) were used for the different analyses, except the sensorial ones. For these last analyses, crackers, biscuits, and cream prepared with the *lpa lec*^−^ flour were compared to the same product prepared without the common bean flour.

For cracker preparation the dough was produced by mixing wheat flour type 2, bean flour, with 95% of water. The dough was left to rest to activate the autolysis process. After 30 min, yeast was added and the remaining 5% of water was gradually added to the dough. The dough was divided in pieces of the same weight and each loaf was reduced to a thin sheet. Each sheet was put in a baking tin, previously oiled, brushed with olive oil and sprinkled with salt to taste. Sheets were pierced and cut in regular pieces and then cooked in an oven at 190 °C for approximately 8 min.

Shortbread biscuits (Biscuit 1) were prepared by mixing sugar with butter in a planetary mixer until they were completely homogenized. Then the other ingredients were added. The dough resulted was soft and biscuits were formed using a sac a poche. Biscuits were cooked in an oven at 190 °C for 14–18 min.

Buckwheat biscuits (Biscuit 2) were prepared by mixing all the ingredients together to produce the dough. Once all the ingredients were completely homogenized, the dough was worked by “cylinder processing” and formed. Before cooking, biscuits were sprinkled with a mixture of sugar or salt and cornmeal. They were cooked in an oven at 190 °C for 14–18 min.

For the cream, the ingredients were mixed together and cooked for approximately 15 min at low temperature (65 °C) to pasteurize the egg yolks. The final cream was stored in the fridge at about 4 °C.

### 2.3. Proximate Composition Analyses

The chemical composition of food samples was assessed according to AOAC standard methods [46]. Samples were milled and analyzed for dry matter (DM), ash, crude protein (CP), and ether extract (EE). Starch content was measured enzymatically (kit K-TSTA, Megazyme, Bray, Ireland). Total sugar determination was performed using the sucrose, D-fructose and D-glucose assay procedure (kit K-SUFRG Megazyme). The total dietary fiber (TDF) was determined using the AOAC Method 991.43 (kit K-TDFR, Megazyme) based on the sequential enzymatic digestion of 1 g of sample by heat-stable α-amylase, protease and amyloglucosidase.

The evaluation of in vitro starch digestion of food products over time was performed following the multi-enzymatic protocol detailed by Giuberti et al. [47] and as described in Sparvoli et al. [12]. Commercial fresh white bread (starch content of 72.3% DM) was used as reference and a blank was also included to correct for the glucose in the amyloglucosidase solution. The percentage of digested starch at each time interval was calculated using a factor of 0.9 to convert mono to polysaccharides. For each treatment, samples were analyzed in duplicate. After the enzyme digestion, a hydrolysis index (HI) was derived from the ratio between the area under the hydrolysis curve (AUHC) of each sample and the corresponding AUHC of the reference fresh white bread as a percentage over the same period. From the obtained HI, a pGI value was calculated with the formula pGI = 8.198 + 0.862 × HI [48].

### 2.4. Hemagglutination Test

Bean flours, or defatted food samples, were extracted with 20 volumes of phosphate buffered saline buffer (PBS: 10 mM KHPO_4_, 15 mM NaCl, pH 7.4). Hemagglutinating activity in the extracts was determined by a serial dilution method using a human type A erythrocyte suspension. Drops of blood were drawn from the donor finger and collected inside 1.5 mL Eppendorf tube: about 150 μL of blood was collected. Erythrocytes were separated from the serum by washing with about 10 volumes (1.5 mL) of PBS. This step was repeated three times. The serum was eliminated, and the erythrocytes were stored at 4 °C in 10 volumes of PBS. At the time of use, erythrocytes were diluted 1:10 with PBS.

For each sample (PBS samples both from flours and defatted products), serial dilutions in PBS, ranging from 1:2 to 1:256, were assayed. Agglutination was visually determined after 4 h incubation at room temperature.

### 2.5. Assay of α-Amylase Inhibitor Activity

The analysis was based on a protocol that measures the inhibitory activity of the sample against human salivary α-amylase (EC 3.2.1.1; Type IX-A) by the increase of iodine staining after the action of the salivary α-amylase with soluble starch [49]. Briefly, different volumes (from 10 to 100 μL) of each sample extract (bean flour or defatted food products in PBS, as described above) were diluted 50 or 200-fold in 20 mM borate buffer pH 9 and were pre-incubated with a fixed amount of α-amylase (0.15 U) for 30 min at room temperature in a final volume reaction of 300 μL. Then, 200 μL of a 0.15% solution of potato starch was added and, after 5 min at room temperature, the reaction was stopped by adding 1ml of iodine reagent [50] and absorbance was measured at 620 nm. Results were expressed as units of α-amylase inhibited per mg of flour, where one unit of inhibitor activity is the amount which will bring about 50% inhibition of the α-amylase in 30 min under the above conditions according to Marshall and Lauda [51]. The percentage of residual activity was calculated comparing the expected U of α-AI/100 mg of flour to the measured values.

### 2.6. Preparation of the Pepsin, Pancreatin and Bile Extract Digests

A quantity of 0.2 g porcine pepsin (800–2500 units/mg protein; Sigma-Adrich, St-Louis, MO, USA) was dissolved in 5 mL 0.1 N HCl; 0.05 g pancreatin (4 × USP specifications, Sigma-Adrich) and 0.3 g bile extract porcine (glycine and taurine conjugates of hyodeoxycholic and other bile salts, Sigma-Adrich) were dissolved in 25 mL 0.1 NaHCO_3_. Both solutions were preliminary subjected to batch treatment with Chelex-100 (Bio-Rad Laboratories, Hercules, CA, USA) to eliminate any residual polyvalent metal ion. A quantity of 1.65 g food sample was dissolved in 10 mL water in a 50-mL screw-cap culture tube. The pH of each sample was adjusted to pH 2.0 with 5.0 N HCl. A volume of 0.5 mL of the pepsin solution was added and incubated for 60 min on a shaker at 37 °C. The pH of the sample was then raised to 6 with 1 M NaHCO_3_ and 2.5 mL of the pancreatin-bile extract mixture was added. Finally, the pH was adjusted to 7 with 2 M NaOH, and the volume was brought to 15 mL with a 120 mM NaCl/5 mM KCl water solution.

### 2.7. In Vitro Analysis of Iron Bioavailability and Iron Content Evaluation

The in vitro digestion model from [52] was adopted with some modifications. Caco-2 cells (American Type Culture Collection (Rockville, MD, USA)) were used in experiments at passage 29. Cells were seeded at densities of 50,000 cells/cm^2^ in collagen-treated 6-well plates (Costar Corp., Cambridge, MA, USA) and cultured at 37 °C in Dulbecco’s modified Eagle medium containing 25 mmol/L HEPES, and 10% fetal bovine serum (complete medium) in 5% CO_2_ atmosphere. On day 13 from cell confluence, the complete medium was removed and replaced with minimum essential medium (MEM) supplemented with 10 mmol/L PIPES, 4 mg/L hydrocortisone, 5 mg/L insulin, 5 µg/L selenium, 34 µg/L triiodothyronine and 20 µg/L epidermal growth factor (enriched MEM), to ensure background levels < 80 µg Fe/L. A Transwell insert ring (Costar Corp.) was then added to each well, in which the upper chamber was formed by fitting the bottom with a dialysis membrane (15,000 Da molecular weight cut off; Sigma-Aldrich). A volume of 1.5 mL digested sample was added to the upper chamber and incubated for 2 h. Then, inserts were removed, and 1 mL of enriched MEM was added. Cells were incubated for a further 22 h at 37 °C. Then, the monolayer was washed with 140 mM NaCl, 5 mM KCl and 10 mM PIPES pH 7 (rinse solution). The rinse solution was aspirated and 2 mL of a freshly prepared rinse solution containing 5 mM sodium hydrosulfite and 1 mM athophenanthroline disulfonic acid (removal solution) was added for 10 min. The removal solution was aspirated and the monolayer was washed with rinse solution. Next, 2 mL of deionized water was added and cells were sonicated for 15 min with a benchtop sonicator. Finally, cells were recovered by scraping, collected along with the 2 mL water and stored at −20 °C. A 10-µL sample was used for ferritin measurement using the Human Ferritin ELISA (Enzyme-Linked Immunosorbent Assay) kit (SIGMA-Aldrich). Cell protein was measured using the Bio-Rad DC protein assay kit (Bio-Rad).

For iron content evaluation, 300 mg samples were digested in Teflon tubes filled with 5 mL of 65% (*v*/*v*) HNO_3_ by a microwave digester system (MULTIWAVE-ECO, Anton Paar Italia Srl., Rivoli, Italy) by applying two-step power ramps (Step 1: To 500 W in 10 min, maintained for 5 min; Step 2: To 1200 W in 10 min, maintained for 15 min). After 20 min cooling, the mineralized samples were transferred into polypropylene test tubes and diluted 1:20 with MILLI-Q water (Merck). Iron concentrations were measured by inductively coupled plasma-mass spectrometry (ICP–MS; Bruker AURORA M90 ICP–MS, Bruker Daltonik GmbH, Leipzig, Germany).

### 2.8. Sensorial Evaluation

**Figure 1:**
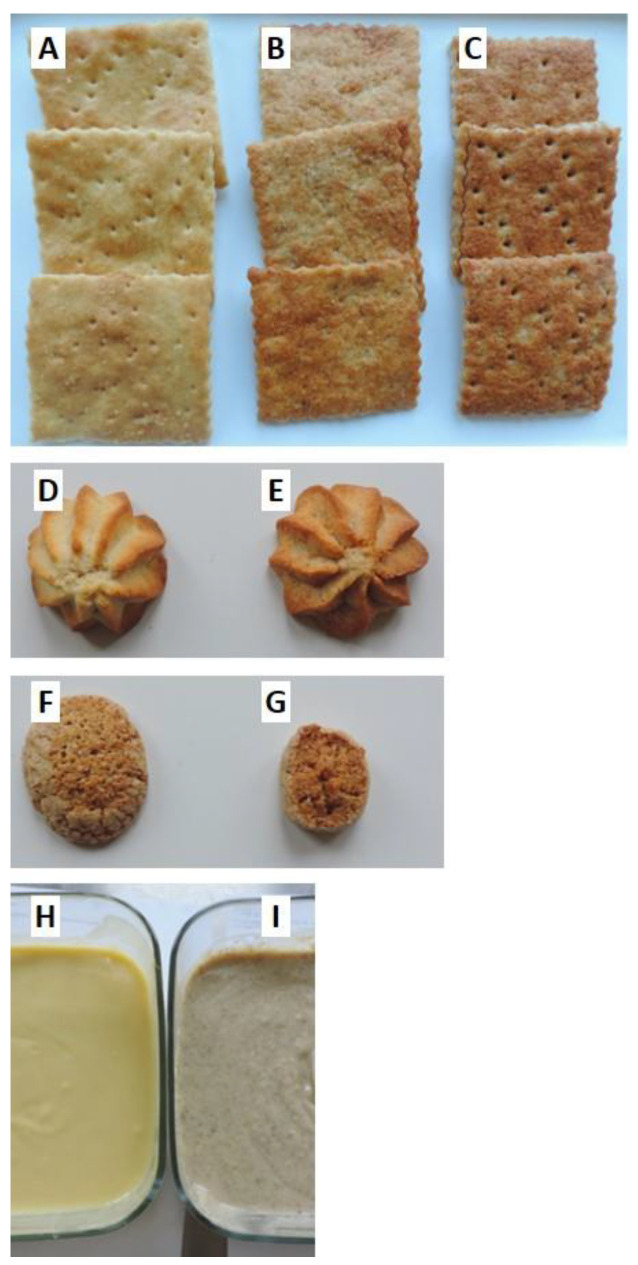
Samples used for the sensorial evaluation. (**A**) Cracker R, (**B**) Cracker 1, (**C**) Cracker 2, (**D**) Biscuit 1R, (**E**) Biscuit 1, (**F**) Biscuit 2R, (**G**) Biscuit 2, (**H**) Cream R, (**I**) Bean Cream. All R samples were reference without common bean flour, while the samples Crackers 1 and 2, Biscuits 1 and 2 and Bean Cream contained 26, 38, 29, 14 and 9% of common bean flour, respectively.

Sensory evaluation was executed on food products prepared with bean flour (Table 1) and on reference I samples, in which bean flour was replaced with equal amounts of wheat flour type 2 (Cracker R, Biscuit 1R), whole wheat flour (Biscuit 2R), or rice flour (Cream R). Evaluated products are shown in Figure 1.

Sensory descriptive analysis (DA) and temporal dominance of sensations (TDS) [53,54], were carried out by a panel composed of 10 expert judges (5 females and 5 males, age 30–50), with significant experience in sensory descriptive evaluation and in the use of the sensorial software used to collect data. Attributes to be used in this study were selected based on literature on bakery goods and cookies [55] and proposed to the judges to familiarize with the products. Tests were performed by the panel at appropriate light and temperature conditions, according to UNI EN ISO 8589:2014 [56], in individual booths, with notebooks equipped with specific software for sensory data acquisition (FIZZ Biosystèmes, France).

Samples were distributed to the judges, coded with three-digit numbers and presented randomly. Mineral water was distributed to the judges to clean their mouths between samples.

#### 2.8.1. Descriptive Analysis

For DA, each judge was delivered one product at a time and asked to evaluate the attributes (Table 2) using a nine-point intensity scale (1 = hardly perceptible; 9 = very intense) an overall quality score (1 = dislike extremely; 9 = like extremely) [57,58]. DA tests were carried out in duplicate.

#### 2.8.2. Temporal Dominance of Sensations

The TDS was performed, considering only 5–7 attributes (Table 2) linked to the gustative/aromatic aspects considered relevant for each product. According to Pineau [53], during the product tasting (60 s) judges were asked to express the dominant attribute.

Results were elaborated by statistical analysis using the software SAS^®^ (SAS 9.4, SAS Institute Inc., Cary, NC, USA). The sensorial profiles were analyzed through ANOVA and post hoc test (Tukey’s HSD) and displayed in a visual plot. For the TDS, the proportion of runs for which each attribute was considered as dominant was calculated for each point in time. These proportions were traced over time using the SAS^®^ TRANSREG procedure and named “TDS curves”.

## 3. Results

### 3.1. Proximate Composition

Crackers and biscuits prepared with wt or *lpa lec*^−^ common bean flours were analyzed for their proximate composition (Table 3). Results showed that the protein content was relatively high in all the baked products, due to the contribution of the bean flour. As expected, the highest protein content was found in Cracker 2 (21.7 g/100 g DM and 18.5 g/100 g DM for *lpa lec*^−^ and wt, respectively), which was characterized by the highest bean flour content (38% of the total product). Biscuits contained the lowest protein content (average 11.3 g/100 g DM), according to the lower percentage of the bean flour in their formulation (20% and 12% of the total in Biscuit 1 and Biscuit 2, respectively).

The lipid content was higher in Biscuit 1 (average 32.4 g/100 g DM) and consistent in Biscuit 2 (average 15.2 g/100 g DM), since, in both cases, butter was one of the main ingredients. In contrast, crackers, which were prepared with no added fats (except for the olive oil used to sprinkle the pan), showed a lower lipid content (average 3.0 g/100 g DM).

The carbohydrate fraction appeared variable between baked products. The starch content was higher in Cracker 1 (average 45.4 g/100 g DM), but was lower in Cracker 2 (average 38.3 g/100 g DM), where the bean flour was present in a higher proportion. In contrast, starch content was higher in Biscuit 1 than in Biscuit 2 (average 20.2 g/100 g DM and 16.7 g/100 g DM, respectively), despite the fact Biscuit 1 contained a higher percentage of common bean flour. This difference might be explained by the composition of Biscuit 2, in which wheat flour was replaced by whole wheat and buckwheat flours that further contributed to lowering the starch content. On the other hand, these ingredients did not influence the content of total dietary fiber (average 16.7 and 17.9 g/100 g). The total dietary fiber also constituted a significant fraction in crackers and increased with the increasing level of bean flour in the products, being higher in Cracker 2 than in Cracker 1 (20.9 g/100 g DM against 16 g/100 g DM). In crackers, total sugars were negligible, while they were relevant in biscuits for the saccharose fraction (glucose and fructose were almost absent) that correlated to the added sugar in the recipes, that was almost three times higher in Biscuit 2 than in Biscuit 1 (average 30.4 g/100 g DM and 12.0 g/100 g DM, respectively).

Finally, the ash content was higher in crackers (average 6.5 g/100 g DM in Cracker 1 and 7.6 g/100 g DM in Cracker 2), while Biscuit 2 had a slightly higher ash fraction (average 1.8 g/100 g DM) than Biscuit 1 (average 1.4 g/100 g). These differences might have been due to the bean flour present in the products, together with the amount of less refined flours in Biscuit 2.

In general, products made with *lpa lec*^−^ bean flour had higher protein content and lower starch content compared to those made with wt bean flour, in agreement with the composition of the respective flours (27.7 g/100 g vs. 23.1 g/100 g for proteins and 36.0 g/100 g vs. 44.9 g/100 g for starch) (Table 3).

### 3.2. Evaluation of Hemagglutinating Activity

**Figure 2:**
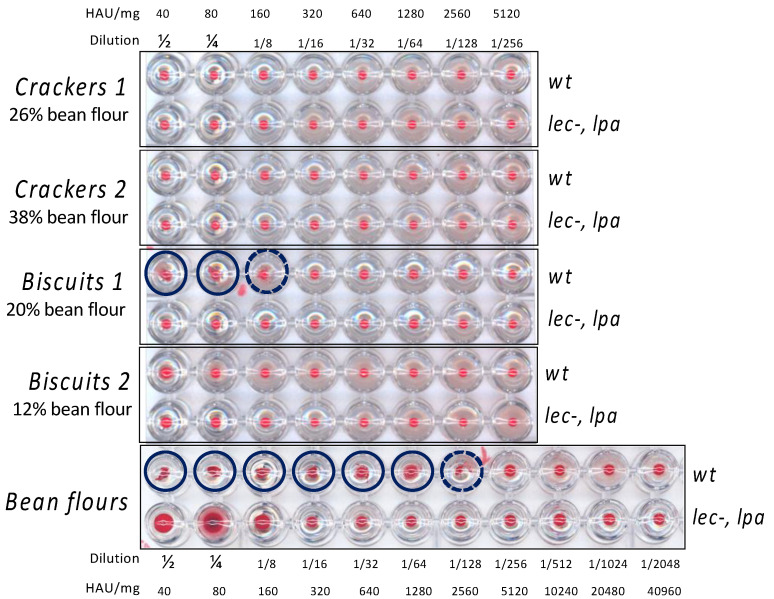
Hemagglutinating activity of bean-based products and bean flour extracts. Serial dilutions of equal amounts of bean-based products or seed flour extracts obtained from two different genotypes (wt and *lpa lec*^−^) were compared. Blue circles indicate sample dilutions able to agglutinate red blood cells. The percentage of bean flour in biscuits was adjusted based on the weight of defatted samples (in defatted biscuits 1 bean flour was 29.4%).

Bean seeds accumulate high amounts of toxic PHAs and the excessive consumption of raw or improperly cooked beans may cause poisoning. Therefore, since our aim was to exploit the use of a biofortified and lectin null bean flour to obtain novel fortified foods, it was important to verify the lack of any residual lectin activity in the bean-based products (crackers and biscuits in their variants). To quantify the amount of lectins in the different bean-based products, equal amounts of extracts were compared with equal amounts of extracts from flours of the wt genotype as control, and *lpa lec*^−^ genotype (Figure 2).

As expected, the flour extract characterized by the absence of active PHAs (*lpa lec*^−^ genotype) was not able to agglutinate blood erythrocytes at any dilution. On the contrary, wt flour extract (containing PHAs) was able to agglutinate blood erythrocytes after a serial dilution until 1/128 (dotted circle in Figure 2).

The hemagglutination test on defatted products (Figure 2) indicated the presence of residual lectin activity only in Biscuit 1 containing 20% wt bean flour (corresponding to 29.4% in defatted flour). Cracker 1 and Cracker 2, containing 26% and 38% of bean flour on the total product, respectively, were not able to agglutinate blood erythrocytes. As expected, none of the products made with *lpa lec*^−^ genotype showed residual hemagglutinating activity. Comparing these agglutination results with those obtained from corresponding amounts of unprocessed bean flours, and considering that defatted Biscuit 1 contains about 1/3 of bean flour, it was possible to quantify the residual PHA activity in processed biscuit 1, prepared with wt common bean flour, being approximately 10–12% that of the corresponding bean flour.

### 3.3. Evaluation of α-Amylase Inhibitor Activity

The α-Amylase inhibitor plays an important role in lowering starch digestion and its effect is reflected in decreased postprandial plasma hyperglycemia and insulin levels, increased resistance of starch to digestion, and increased activity of the colorectal tract, as well as reduction in the glycemic index [12,59]. Processed products characterized by total, or at least partial, α-AI activity retention could be introduced in diets for people who require a low blood-glucose level (including individuals with type 1 diabetes) for the maintenance of good health status. To verify the stability and residual activity of α-AI in different processed products, α-AI activity was assessed in defatted extracts of crackers, biscuits and lyophilized cream, and compared to the α-AI activity of control raw flours.

Results of α-AI activity of tested products are reported in Table 4. An insignificant residual inhibitory activity was detected in Crackers 1 and 2 when made with wt bean flour, while a higher percentage of residual activity was reported for crackers made with *lpa lec*^−^ genotype (16.63% and 32.34% respectively for crackers 1 and 2). Biscuit 1 was characterized by limited retention of α-AI activity when made with wt bean flour (20.44%), while biscuits made with the flour from the *lpa lec*^−^ genotype showed higher inhibitory activity (51.19%). Biscuit 2 was characterized by higher residual α-AI activity compared to Biscuit 1, however it behaved in a similar way as regards the selected genotype for the bean flour; when *lpa lec*^−^ bean flour was used, the residual α-AI activity was more than double that of Biscuit 2 containing wt bean flour (84.17% for *lpa lec*^−^ and 37.33% for wt). In the case of the cream a moderate retention of α-AI activity was observed (27.28%). This product was made only with the *lpa lec*^−^ bean flour as, if using wt flour, a high lectin activity would be expected due to the mild cooking conditions (70 °C, see Section 2).

Interestingly, in all samples extremely high residual α-AI activity was always detected when the *lpa lec*^−^ bean flour was used instead of the wt genotype and the highest activity was found in Biscuit 2, although it was not the one with the highest proportion of bean flour, suggesting that most probably, the food matrix and the cooking conditions are important for maintenance of α-AI stability and activity.

### 3.4. In Vitro Predicted Glycemic Index

The predicted in vitro glycemic index (pGI) was analyzed for crackers and biscuits using common white bread as reference. Results are reported in Table 5, where pGI was compared in the carbohydrate (starch and saccharose) fractions of each sample.

Considering the different bean-based products, Cracker 1 had the highest pGI (average 74), which correlates to its high percentage of starch (average 45.4 g/100 g), primarily deriving from wheat flour. In contrast, in Cracker 2, the higher amount of bean flour (38% in Cracker 2 vs. 26% in Cracker 1) contributed to reducing the starch fraction (37.5 vs. 45.4 g/100 g) and to increasing the dietary fiber and the crude protein contents, thus lowering the pGI value.

The biscuits had a more complex composition and a similar pGI (average 41.3 for biscuits 1 and 47.7 for biscuits 2). The starch fraction was higher in Biscuit 1 (20.2 g/100 g vs. 16.7 g/100 g), while sugars, consisting almost entirely of saccharose, were more than doubled in Biscuit 2 (30.4 g/100 g vs. 12.0 g/100 g). Saccharose was thus primarily responsible for the higher pGI of Biscuit 2. Biscuit 2 was also characterized by the presence of whole wheat and buckwheat flours that counterbalanced the effect of sugars by contributing to an increased total fiber fraction, which was quite similar to that of Biscuit 1 (17.9 g/100 g vs. 16.6 g/100 g) in which a major contribution derived from bean flour, which also contributed, together with a reduced saccharose content, to the slight reduction of pGI.

Interestingly, if data were compared considering the contribution of the bean genotype, it clearly appeared that, when products contained the *lpa lec*^−^ bean flour, a reduction of about 5 units of the pGI, corresponding to about 7% of the reference (white bread) pGI; was detected (71.4 vs. 76.6 for Cracker 1, 55.4 vs. 61.6 for Cracker 2, 39.8 vs. 42.9 for Biscuit 1, 45.0 vs. 50.4 for Biscuit 2).

### 3.5. Assessment of Fe Bioavailability of Bean Biofortified Biscuit 1 in Caco-2 Cells

**Figure 3:**
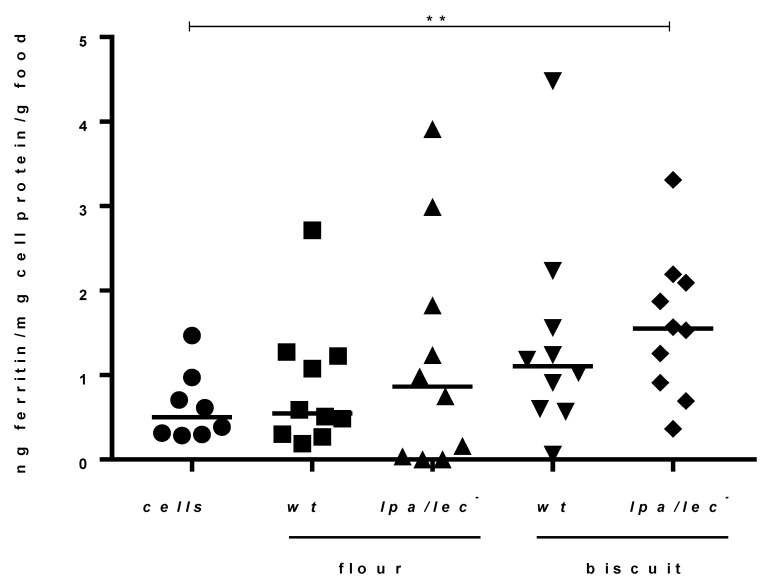
Ferritin production after incubation of Caco-2 cells with wild type (wt) flour, *lpa lec*^−^ flour, wt Biscuit 1 or *lpa lec*^−^ Biscuit1 samples. Samples (n = 10) were statistically evaluated by Mann-Witney test. ** *p* = 0.085.

We determined the available iron in examined food samples by analyzing ferritin formation in the intestinal cell line Caco-2. This parameter is considered a good indicator of cell Fe uptake from the food digest [52,60]. Caco-2 cell ferritin formation was shown to increase linearly in the range 50–100 µM Fe [52]. Accordingly, based on our estimated of Fe content (Table 6), 1.65 g was identified as an appropriate amount of food to digest, in order to maintain the Fe concentration within the 50–100 µM range (Fe molar mass: 55.845 g/mol). Our analysis showed very low levels of ferritin formation in untreated Caco-2 cells (Figure 3), in agreement with previous data [52]. The test was characterized by high sensitivity because it also allowed estimation of the small variations recorded among all the examined samples. On the other hand, following challenge with food sample digests, the resulting ferritin production was quite variable and required an appropriate number of biological replicates (n = 10) to detect any trend or achieve statistical significance. Interestingly, both *lpa lec*^−^ bean and Biscuit 1 flours tended to have a higher ferritin value than their wt counterpart. In addition, ferritin levels in *lpa lec*^−^ Biscuit 1 were significantly higher compared to cells alone. (Figure 3).

### 3.6. Sensory Analyses of Bean-Based Products

**Figure 4:**
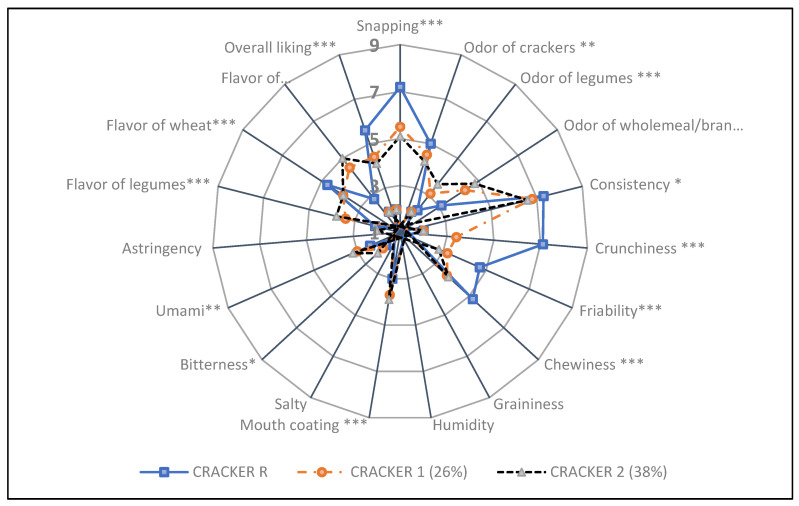
Sensorial profiles of crackers with [Cracker 1 (26%); Cracker 2 (38%)] and without [Cracker R] bean flour. Data analyzed by ANOVA and post hoc test (Tukey’s HSD); *, **, *** indicate significant differences between the tested products at *p* < 0.1, *p* < 0.5 and *p* < 0.01, respectively.

Crackers 1 and 2 had very similar characteristics, differing only for crunchiness, higher in Cracker 1. On the other hand, both crackers significantly differed from the control product with lower snapping, crunchiness, friability, chewiness and wheat flavor, and higher intensities of legume, wholemeal/bran odor and flavor, cracker odor, mouth coating and umami. In addition, Cracker 2 was also different from the reference for lower cracker odor and firmness, and higher intensity of bitterness. Both Crackers 1 and 2 recorded an acceptance score (4.4 and 4.1, respectively) between “Neither Like nor Dislike” and “Dislike Slightly”, corresponding, respectively, to 79% and 73% of the score obtained by the Cracker R (5.6) (Figure 4).

**Figure 5:**
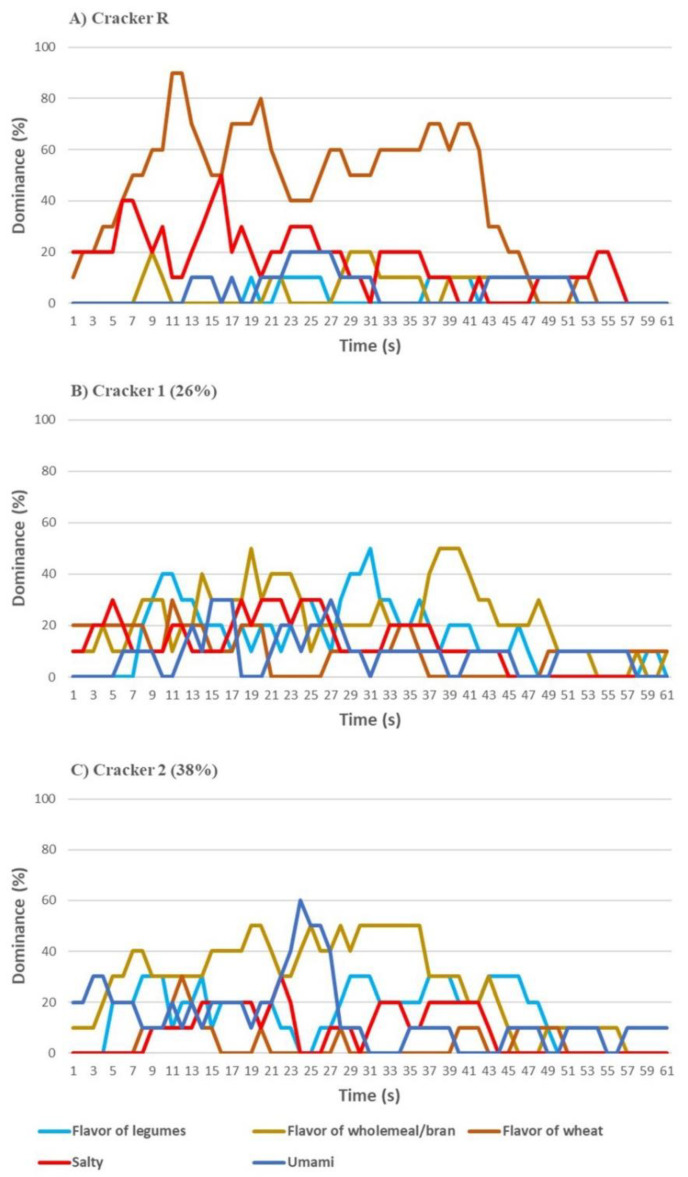
Temporal dominance of sensations (TDS) flavor of (**A**) Crackers R (reference, without common bean flour), (**B**) Crackers 1 (with 26% common bean flour) and (**C**) Crackers 2 (with 38% common bean flour).

Regarding the TDS analysis for flavor, in Cracker R (0% bean flour) the flavor of wheat was predominant over the whole tasting; salty showed an important role in the first phase of tasting (Figure 5A). With the addition of 26% bean flour, the flavor of legumes and whole-wheat became dominant (Figure 5B); a further addition of bean flour increased the dominance of whole-wheat flavor, together with a clear perception of umami in the central part of the tasting course (Figure 5C).

**Figure 6:**
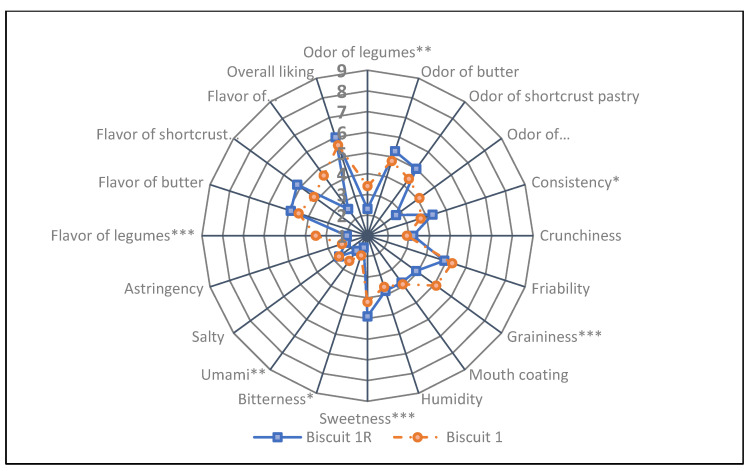
Sensorial profile of shortbread biscuits without (Biscuits 1R, reference) and with common bean flour (Biscuit 1). Data analyzed by ANOVA and post hoc test (Tukey’s HSD); *, **, *** indicate significant differences between the tested products at *p* < 0.1, *p* < 0.5 and *p* < 0.01, respectively.

Biscuit 1 had a more intense odor and flavor of legumes and wholemeal/bran, as compared to Biscuit 1R. Moreover, Biscuit 1 was perceived as grainier, bitter and with a higher intensity of umami. The control biscuit (Biscuit 1R) was perceived as more consistent, sweeter with a stronger shortcrust pastry flavor (Figure 6). Both biscuits recorded an overall liking over “Neither Like nor Dislike”, with Biscuit 1 recording an overall liking of 5.6, corresponding to 93% of the reference biscuit score.

**Figure 7:**
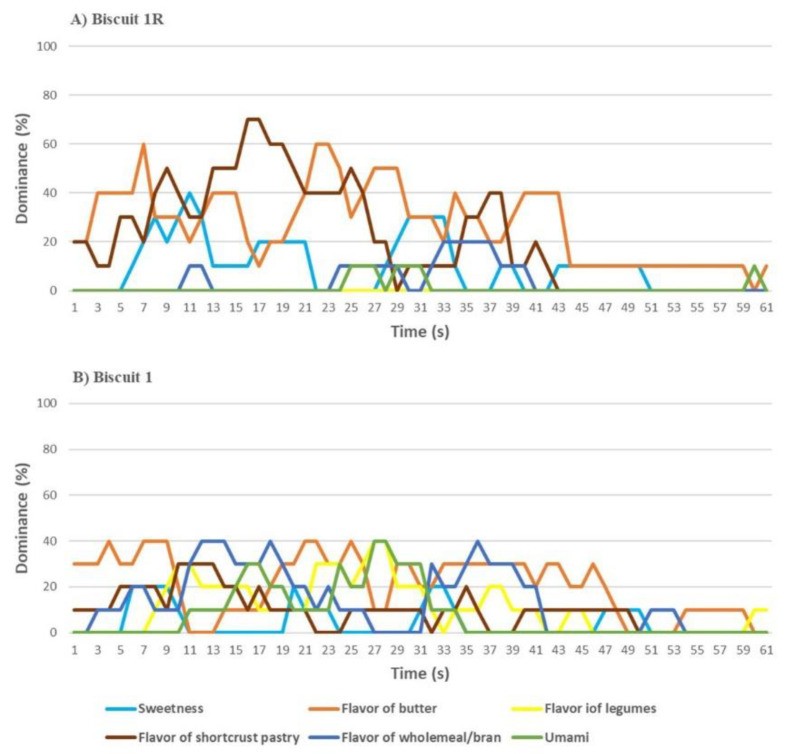
Temporal dominance of sensations (TDS) of (**A**) Biscuits 1R (reference, without common bean flour) and (**B**) Biscuit 1 (with 29% common bean flour).

The TDS of Biscuit 1R (Figure 7A) was mainly characterized by butter flavor and of shortcrust pastry, being the first perceived during the first and last part of tasting, and the second having higher impact in the middle part. In Biscuit 1 (Figure 7B), the flavor of wholemeal/bran and butter alternated for dominance during the tasting. Umami and flavor of legumes increased from the beginning to the central segment of tasting where they were the most perceived.

**Figure 8:**
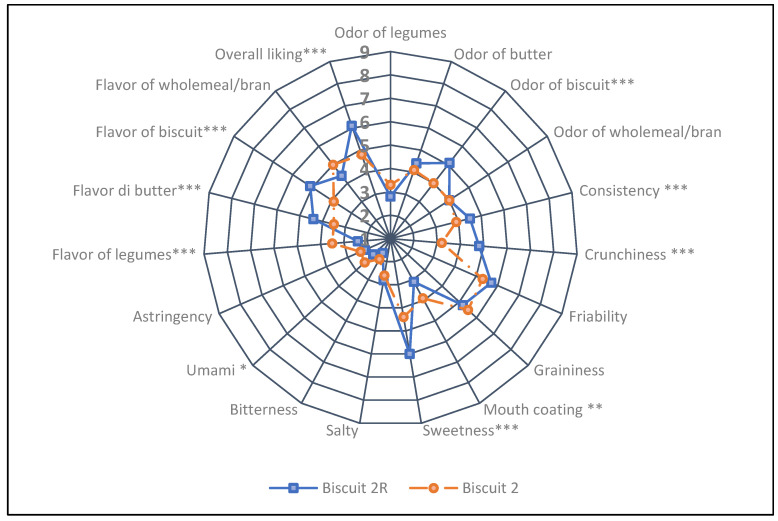
Sensorial profiles of type 2 Biscuits, without (2R) and with common bean flour (2). Data analyzed by ANOVA and post hoc test (Tukey’s HSD); *, **, *** indicate significant differences between the tested products at *p* < 0.1, *p* < 0.5 and *p* < 0.01, respectively.

Biscuit 2, as compared to its reference, had a higher mouth coating, while the umami taste was perceived more, as well as the flavor of legumes. The Biscuit 2R showed a higher biscuit odor, consistency and crunchiness; it was perceived to be sweeter than Biscuit 2 and with a more intense flavor of butter and biscuit (Figure 8). A significant difference was recorded for overall liking: Biscuit 2R recorded a score of 6.1, superior to the “Like Slightly” level. On the other hand, Biscuit 2 was rated between “Dislike Slightly” and “Neither Like not Dislike”, despite recording an overall liking of 4.8, corresponding to 79% of the reference biscuit score.

**Figure 9:**
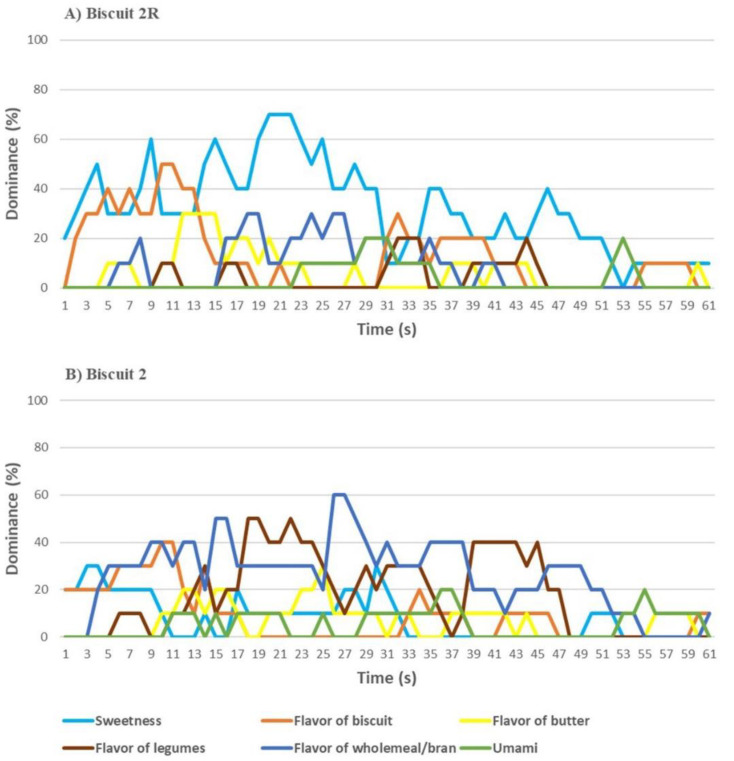
Temporal dominance of sensations (TDS) of (**A**) Biscuits 2R (reference, without common bean flour) and (**B**) Biscuit 2 (with 14% common bean flour).

The TDS of biscuit 2R with 0% bean flour (Figure 9A) showed a prevalence of flavor of shortcrust pastry and butter during the whole tasting. In the biscuit with 20% bean flour (Figure 9B), the flavor of wholemeal/bran and butter alternated during the tasting (the latter with a lower dominance than in the biscuit without bean flour). Umami and flavor of legumes increased from the beginning to the central segment of tasting where they were the most perceived.

Regarding the TDS flavor of Biscuit 2 (Figure 9A), the sweetness dominated during almost the whole tasting, with the flavor of biscuits being more perceived mainly in theinitial phase. In Biscuit 2 sweetness was dominant at the very beginning of tasting; the wholemeal/bran and legumes flavor alternated in dominance during the remaining tasting time. A slight umami peak was recorded at the end (55–60 s).

**Figure 10:**
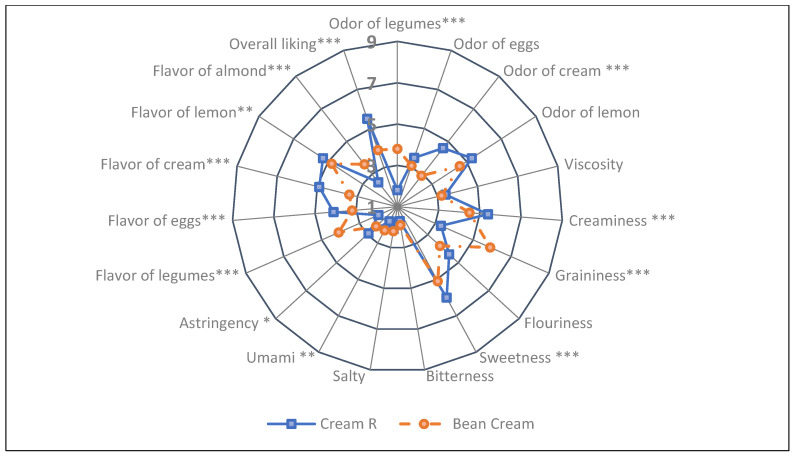
Sensorial profile of cream without (Cream R, reference) and with common bean flour (Bean Cream). Data analyzed by ANOVA and post hoc test (Tukey’s HSD; *, **, *** indicate significant differences between the tested products at *p* < 0.1, *p* < 0.5 and *p* < 0.01, respectively.

Regarding the two cream preparations it was possible to observe several significant differences. In particular, the bean flour Cream had higher odor and flavor of legumes and flavor of almond. Moreover, it was rated as grainier and more sapid (i.e., high umami perception) (Figure 10). The Cream R preparations differed also, for more perceived cream odor and flavor of eggs, cream and lemon. Creaminess and sweetness were higher in Cream R as well as astringency. The overall liking of Cream R was between “Neither Like not Dislike”, and “Like Slightly”, while bean Cream was below the “Dislike Slightly” point of acceptability, recording an overall liking of 3.9, corresponding to 71% of the reference biscuit score.

**Figure 11:**
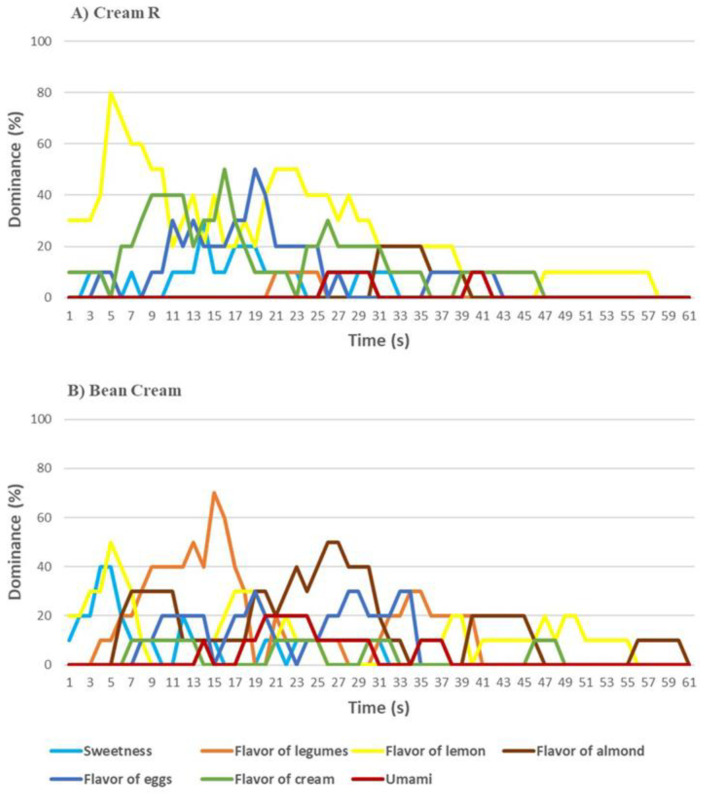
Temporal dominance of sensations (TDS) of (**A**) Cream R (reference, without common bean flour) and (**B**) Bean Cream (with 9% common bean flour).

The TDS taste of Cream R (Figure 11A) showed a dominant peak of lemon flavor at the beginning of the tasting (around 11 s), followed by the flavor of cream and eggs, after that the impact of lemon was noteworthy, since it was the sensation that dominated during the remaining tasting time. The bean cream tasting was characterized by lemon flavor and sweetness at the beginning, which were substituted by the flavor of legumes and, later, the flavor of almond (Figure 11B). The last part of tasting was characterized by the flavor of lemon and almond.

## 4. Discussion

The nutritional quality of traditional snacks is often considered low due to their high sugar and fat content and their protein deficiency [61]. However, for many people they constitute a considerable fraction of daily calories, representing a fast and convenient food source that can be consumed without preparation [62]. In this scenario, healthier food snacks are required to respond to an increasing necessity by consumers to consume healthier snacks. Crackers and biscuits are among the most widely available baked goods used as snacks as they combine nutrition, long shelf-life and practicality. Following the footsteps of our previous study [12], with the present work, we contributed with new knowledge and genetic materials useful to the development of nutritionally improved and healthier snack foods (e.g., crackers, biscuits and a cream) based on a biofortified and low antinutrient common bean flour. Furthermore, data on the sensorial characteristics of these products provided indications to better identify target consumers as well as to develop bean-based products better adapted to consumer needs. From a nutritional standpoint, the use of composite flours containing common bean flour led, as expected and reported in similar studies [7,12,15,63,64], to products with higher protein and total dietary fiber content and lower amounts of starch and total carbohydrates. Depending on the type of pulse-based snacks (e.g., bars, biscuits, chips, bread, extruded snacks) reported protein concentration has ranged between 8.79% and 29.8%, with extruded snacks having the highest protein content, as they mostly or exclusively contain legume flour [7,64]. The direct comparison between the two types of biscuits produced in the current study with other bean-based biscuits previously described [12,15] showed that the protein content of Biscuit 1 and Biscuit 2 was higher (by on average 11%) than that (ranging between 8% and 10%) reported for biscuits containing bean flour ranging from 12% to 29%, indicating that the composition of the composite flour is also important if other flours beside common bean and wheat are used. In fact, the use of maize flour depleted the protein content of the products [12,15], while the addition of buckwheat flour, together with the presence of eggs, increased it in Biscuit 2 (12% of bean flour) compared to Biscuit 1 (20% bean flour). This was also reflected in starch content which was lower in Biscuit 1 and Biscuit 2 (19.3% and 14.8%, respectively) compared to that of biscuits containing from 12% to 29% bean flour (31.1% and 26.4%, respectively) and control biscuits (41.9%) [12].

If an increase in protein and total dietary fiber and a decrease in starch contents in the bean-based products could be expected, the major element of novelty of the present study relates to the fact that the bean-based products were obtained using flours of biofortified and low antinutrient (*lpa lec*^−^) bean genotypes. Common approaches to increase essential mineral bioavailability and to reduce antinutrients are mainly based on a combination of technological processing, such as soaking, dehulling, heating, cooking, fermentation, germination and extrusion [7]. These treatments, although necessary to reduce or eliminate the antinutrients, are costly and time consuming and may reduce the nutritional quality of the product (e.g., loss of minerals in brine). In contrast, bean genotypes carrying the *lpa* and *lec*^−^ mutations do not need specific treatments, such as soaking, to reduce phytic acid or heating of the flour to inactivate the lectins, as demonstrated and discussed in this study and in a previous one [12].

Iron deficiency is a serious issue in many areas of the world. Nearly two billion people are currently iron-deficient, especially resource-poor women, infants, and children in developing countries [65]. The common bean is a strategic crop for biofortification and it has been included in breeding programs in the HarvestPlus international research program [66] supporting the research and development of biofortified crops. Although most of the research has been aimed at increasing the amount of iron in the seed, now it is quite clear that the most powerful approach should also consider the reduction of phytic acid, to ensure iron bioavailability [67]. Indeed, a number of studies have demonstrated that the *lpa* bean mutant is able to provide more bioavailable iron both by using a Caco2-cells model system [60] and by performing clinical studies on volunteer women [27,44]. However, no data are available on the evaluation of the *lpa* trait on the iron bioavailability inside processed snacks. Here, it was shown that biscuits made with the *lpa lec*^−^ bean flour allow iron to be more bioavailable.

Lectins, and in particular common bean PHA, may exert a very toxic action on the consumer if not properly heat inactivated, as shown by Petry et al. (2016) who observed unexpected adverse gastrointestinal symptoms in Rwandese women with low iron status participating in a clinical trial aimed at evaluating the biofortification potential of *lpa* beans. In a recent work, Cominelli et al. [45] demonstrated that these unwanted effects were due to the role played by the *lpa* mutation on the thermal stability of PHA-L, while no significant effects were observed on the thermal stability of PHA-E or PHA-E and PHA-L oligomers. The role of baking on PHA activity was assessed by Sparvoli et al. [12], which demonstrated that, after baking, sample extracts were still partially able to agglutinate erythrocytes, but this activity was significantly decreased with only three minutes of overbaking. Their biscuits retained about 5–10% PHA activity. Our results confirmed the previous finding, showing that no agglutination activity was detected in *lpa lec*^−^ bean flour and its derivative products. At the same time, a very low residual agglutinating activity was found in bean-based baked products containing active PHA. This activity was higher in the *lpa* genotype for both types of biscuits (about 20–25%) than in the wt genotype Biscuit 1 (about 10–12%). In both Biscuits 1 and 2, the *lpa* genotype retained a more pronounced activity that correlated with higher agglutinating activity of the seed flour. Due to the more efficient heat penetration that allowed complete PHA inactivation, the crackers did not show any residual agglutination activity for all the used common bean genotypes, confirming that proper heat treatment is necessary to obtain a significant reduction or complete inactivation of the toxic protein fraction in PHA-containing genotypes.

In contrast to what was observed for PHA, when the residual α-AI was assayed, all samples containing the *lpa lec*^−^ bean flour showed higher activity than that measured in samples made with wt bean flour. This finding was not completely unexpected and confirmed the findings of a previous study in which it was observed that B14 biscuits made with Lady Joy bean flour (the parent used to introgress the *lec*^−^ and α-AI traits in the *lpa lec*^−^ genotype) showed higher α-AI activity than that of B14 biscuits made with wt bean flours of Taylor and Billò genotypes [12]. Since the α-AI activity measured in the *lpa lec*^−^ flour was lower than that of the wt, these data, together with the above observations, strongly suggest that the α-AI associated to the *lec*^−^ trait should be particularly resistant to thermal denaturation and deserves further study to understand the basis for its behavior.

Legume flours are highly recommended to decrease the glycemic index as they are rich in resistant starch and have a high dietary fiber content. For instance, lupin-based biscuits snacks offered to Type 2 diabetes mellitus (T2DM) patients suggested they may improve both their glycemic control and satiety and a number of clinical trials have been conducted, determining the postprandial glycemic response and the pGI of snacks enriched with legume flours [64,68]. Theoretically, foods can be categorized into low (<55), medium (55–69), and high glycemic index (>70) [69]. Based on their pGI values, the two biscuit types (pGI 41.3 and 41.7) and Cracker 2 (pGI 58.5) can be classified as low and medium pGI snacks, respectively, while Cracker 1 (pGI 74), which contained a smaller amount of bean flour, can be classified as high glycemic index (Table 5). These pGI values are, on average, lower than those reported for other bean biscuits, which ranged between 60 and 80 [12], and are in line with those reported for legume-enriched biscuits (reviewed by Binou et al. [64], indicating that the new formulations proposed in this study have an improved nutritional impact (indeed, they also have better protein and starch content). Interestingly, all the products made with the *lpa lec*^−^ bean flour showed pGI reductions of about 5 units compared to those of corresponding products made with wt flour. The presence of active α-AI, the activity of which was particularly pronounced in baked products made with *lpa lec*^−^ bean flour, is most likely the reason for the observed reduced pGI values. Indeed, B14 biscuits made with bean flour devoid of α-AI showed a pGI about 4–6 units higher than that of the same product made with a wt bean flour [12]. In a very recent study, a bean-based biscuit containing 25% of common bean flour, and with a composition very similar to that of the B24 biscuit of Sparvoli et al. [12], has been shown to be able to reduce the glycemic response and to increase satiety perception [15]. Thus, it can be argued that the low pGIs observed for the *lpa lec*^−^ bean products here described should be predictive of hypoglycemic functional properties. A first step toward the expansion of the legume-based snacks market to more specific consumers’ sectors is to ensure their sensorial appreciation by target consumers. The incorporation of legumes into various products usually results in “beany” flavor/back notes, which are considered “unpleasant” by some people. Furthermore, fortification of cereal-based products with legume flours affects the texture of the end-product [64]. In this study, the Biscuit 1 acceptance score was 93% of that of the control and it also received a better score for all the textural attributes, including crunchiness, consistency and friability. The other bean-based products were less appreciated than traditional ones (reference, R), having an acceptance score ranging between “Neither Like nor Dislike” and “Dislike Slightly”, corresponding to a range between 70–79% that of the control. Crackers 1 and 2 were the least appreciated due their lack of friability and crunchiness, typical of traditional crackers, but also due to the presence of excessive humidity, adhesiveness and chewiness. Even in the case of the creams, the one containing bean flour was characterized by a worse texture, consisting of a higher graininess and poor creaminess. In contrast, among the bean-based products, the biscuits seemed to have the best textural attributes and a consistency that was comparable to that of traditional ones. Moreover, judges agreed in underlining the dominant flavor and taste of legumes in bean-based crackers (especially in Cracker 2), which was intense, even if to a lesser extent than in biscuits and creams. This property may be not appreciated by typical consumers, because it was also accompanied by a pronounced astringency and bitterness. These results are consistent with those of other studies. In Sparvoli et al. [12] liking scores of bean-based biscuits decreased with increase in the bean flour content. The sensory evaluation of gluten-free biscuits, based on different formulations of cereals with the addition of chickpea or lentil flour, showed that the control biscuit had the best liking score, and the other pulse-based products ranged between “Slightly Pleasant” and “Slightly Unpleasant”. Despite the liking scores, they were all considered satisfying as only one sample received the “Slightly Unpleasant” evaluation [70]. Moderate to low texture scores were recorded in a study based on the sensorial evaluation of bean-based, nutrient-enriched, puffed snacks by [71], according to which this characteristic could be attributed to the protein-starch interactions or the starch-fiber interactions that tend to limit expansion of extrudates. Regarding flavor attributes, [72,73] low flavor scores were also associated with the inherent beany flavors, not desired by consumers.

Interestingly, all the bean-based products demonstrated an intense umami taste. Umami, which is also described as the fifth taste, stimulates salivation, improves hypogeusia by enhancing the gustatory-salivary reflex, enhances appetite and satiety, ameliorates eating disorders, and increases the peristaltic reflex and pellet propulsion through the distal colon [74]. This is regarded as a positive attribute, particularly for older consumers, whose senses of taste and smell may have decreased over time; umami could enhance appetite and, as a result, health of tissues like bones and muscles, thus contributing to the maintenance of good health in the elderly [75]. Furthermore, it has been shown that umami compounds significantly attenuate the sensitivity to sucrose, due to competition for binding to the common receptor subunit T1R3 [76] and this might explain the finding that reduced sweetness was perceived for all the bean-based sweet products (Biscuits 1 and 2 and Cream).

These results, although encouraging, also underlie the need to modify the product formulations (especially that of crackers, which require significant enhancement) and to improve their technological, functional and sensorial properties. To this end, sourdough fermentation (by lactic acid bacteria) technology looks very promising as it may improve texture and flavor characteristics, as well as nutritional quality. The use of this type of fermentation on *lec*^−^ bean flour drastically reduced the phytic acid content, increased antioxidant capacity and, most interestingly, did not affect α-AI activity [77]. Therefore, it would be interesting, in future, to apply such technology to produce bean flours to be used for bakery products. Another possibility to improve sensorial properties is to use flours obtained from cooked legumes. In fact, in a study in which sensory analysis of gluten-free rice and common bean biscuits were performed, it has been shown that the most disliked biscuits were those made with raw bean flour, while those made with cooked bean flour received a better acceptance score [78]. Furthermore, it is well known that heat treatment of legume flours may control off-flavors, and hence improve the sensory quality of baked products [79].

The acceptance of bean-based products by the consumer is important to embrace the possibility of extending their market to a more diverse and wider group of people. Beyond their interesting use in feeding of children, bean-based products can be offered to elderly people, as well as successfully employed as efficient substitutes in diets for people suffering from celiac disease, diabetes and obesity. It is quite common that products characterized by the presence of unusual ingredients, including whole wheat, buckwheat and bean flours, tend to have a lower degree of acceptability (due to e.g., high level food neophobia of some consumers) [80]. However, usually their perception from consumers changes when these products are related to a detailed description of their health effects. People are more likely to appreciate uncommon foods when they are sure to receive a positive effect on the body from their regular consumption. For this reason, it would be useful to perform a deeper analysis in which the evaluation also considers this aspect, to understand the real willingness to buy these products by target consumers, considering too that children have a different approach to products from adults. Even teenagers have a different perception of product quality from their parents [81] and this will ultimately lead to different hedonic evaluations and different optimal product formulation.

## 5. Conclusions

In the present study, it was demonstrated that the use of a biofortified and lectin free bean flour (*lpa lec*^−^), instead of that from a wt bean genotype, further improved the nutritional properties of different products. The *lpa lec*^−^ bean flour guaranteed the absence of any possible poisoning due to lectins and provided more bioavailable iron, as demonstrated by using a Caco-2 cells model system. All the products made with the *lpa lec*^−^ bean flour retained higher α-AI activities and had lower pGIs. It would be worthwhile in the future to evaluate the impact of such snacks, particularly those with the lowest pGI and the highest α-AI activity, on the glycemic response, weight gain, and appetite control using an animal model system, thus providing evidence of the health benefits of such products as has been performed for other types of snacks [82].

These products may meet the nutritional needs of children and elderly people well, as they provide more bioavailable iron, are more protein-rich, have lower pGIs, and their umami taste may enhance appetite and satiety, ameliorating eating disorders.

Although these results are encouraging, there remains a need to improve the technological, functional and sensorial properties of the products and, most importantly, to evaluate the extent of these improvements in the light of how consumer perception may change with information about the nutritional and functional advantages derived from consuming the bean-based products.

## Figures and Tables

**Table 1:** Formulations of bean-based food products.

Product	Ingredients	Quantity (g)	% Total	% Bean Flour in	
				Total Flour	Whole Product
Cracker 1	Wheat flour type 2 *	600	38.10	40	26
	Bean flour	400	25.40		
	Water	550	34.92		
	Yeast	10	0.63		
	Salt	15	0.95		
Cracker 2	Wheat flour type 2 *	400	24.62	60	38
	Bean flour	600	36.92		
	Water	600	36.92		
	Salt	15	0.92		
	Yeast	10	0.62		
Biscuit 1 (shortbread)	Butter	500	30.30	50	20
	Milk	350	21.21		
	Wheat flour type 2 *	325	19.70		
	Bean flour	325	19.70		
	Vanilla icing sugar	150	9.09		
Biscuit 2 (buckwheat biscuit)	Sugar	350	27.08	33	12
	Eggs	250	19.34		
	Butter	200	15.47		
	Whole wheat flour *	160	12.38		
	Buckwheat flour	160	12.38		
	Bean flour	160	12.38		
	Backing	12.5	0.97		
Cream	Almond milk	1000	28.41	100	9
	Sugar	300	8.52		
	Eggs yolks	300	8.52		
	Bean flour *	150	4.26		
	Lemon peel	10	10		

* in control products equivalent amounts of these flours replaced the common bean flour. In the case of the cream, rice flour replaced bean flour.

**Table 2:** List of attributes evaluated in descriptive analysis (DA) and temporal dominance of sensations (TDS).

	Cracker	Biscuit 1	Biscuit 2	Cream
Flavor of legumes	DA + TDS	DA + TDS	DA + TDS	DA + TDS
Flavor of wholemeal/bran	DA + TDS	DA + TDS	DA + TDS	
Flavor of wheat	DA + TDS			
Flavor of butter		DA + TDS	DA + TDS	
Flavor of shortcrust pastry		DA + TDS		
Flavor of biscuit			DA + TDS	
Flavor of almond				DA + TDS
Flavor of lemon				DA + TDS
Flavor of cream				DA + TDS
Flavor of eggs				DA + TDS
Flavor of biscuit				DA
Odor of legumes	DA	DA	DA	DA
Odor of wholemeal/bran	DA	DA	DA	
Odor of butter			DA	
Odor of shortcrust pastry		DA		
Odor of crackers	DA			
Odor of biscuit			DA	
Odor of eggs				DA
Odor of cream				DA
Odor of lemon				DA
Snapping	DA			
Consistency	DA	DA	DA	
Crunchiness	DA	DA	DA	
Friability	DA	DA	DA	
Graininess	DA	DA	DA	DA
Viscosity				DA
Creaminess				DA
Adhesiveness	DA	DA	DA	
Humidity	DA	DA		
Chewiness	DA			
Flouriness				DA
Astringency	DA	DA	DA	DA
Umami	DA + TDS	DA + TDS	DA + TDS	DA + TDS
Bitterness	DA	DA	DA	DA
Salty	DA + TDS	DA	DA	DA
Sweetness		DA + TDS	DA + TDS	DA + TDS

**Table 3:** Proximate composition of bean-based products (g/100 g dry matter). na, non-analyzed; <LOQ, under limit of quantification.

Sample		Water	Crude Protein	Crude Lipid	Total Carbohydrates					Ash
					Starch	Saccharose	Glucose	Fructose	Total Dietary Fiber	
Cracker 1	*lpa lec* ^−^	7.3	19.1	3.3	44.6	<LOQ	0.9	<LOQ	16.7	6.3
	wt	6.3	17.0	3.1	46.3	<LOQ	0.9	<LOQ	17.9	6.7
Cracker 2	*lpa lec* ^−^	6.5	21.7	3.4	36.6	<LOQ	<LOQ	1.0	21.7	7.5
	wt	6.9	18.5	2.2	38.5	<LOQ	1.1	0.5	22.8	7.8
Biscuit 1	*lpa lec* ^−^	4.6	11.7	32.0	19.3	11.3	0.0	<LOQ	18.4	1.3
	wt	5.0	11.0	32.9	21.2	12.7	0.0	0.0	14.8	1.5
Biscuit 2	*lpa lec* ^−^	4.6	11.6	15.4	14.8	32.3	0.1	<LOQ	18.2	1.8
	wt	4.5	11.0	15.0	18.6	28.5	0.0	<LOQ	17.7	1.8
Bean flours	*lpa lec* ^−^	10.6	27.7	0.6	36.0	na	na	na	na	3.5
	wt	10.6	23.1	1.1	44.9	na	na	na	na	3.8

**Table 4:** α-amylase inhibitor activity (%) in defatted bean-based product extracts. U α-AI, Units of α-amylase inhibitor.

Sample		% Bean Flour in the Total Sample	Expected U α-AI/100 mg Flour	Measured U α-AI/100 mg Flour	% Residual α-AI Activity
Control flours	wt	100	-	1552.33	-
	*lpa lec* ^−^		-	1253.10	-
Cracker 1	wt	26	620.93	4.23	0.68
	*lpa lec* ^−^		501.24	83.86	16.63
Cracker 2	wt	38	931.40	2.20	0.24
	*lpa lec* ^−^		640.35	207.09	32.34
Biscuit 1	wt	29 ^a^	494.73	101.11	20.44
	*lpa lec* ^−^		399.36	204.43	51.19
Biscuit 2	wt	14 ^a^	232.38	87.77	37.77
	*lpa lec* ^−^		187.60	157.89	84.17
Cream	*lpa lec* ^−^	24 ^b^	304.50	83.06	27.28

^a^ percentage adjusted on the weight of defatted samples; ^b^ percentage adjusted on the weight of the lyophilized sample.

**Table 5:** In vitro predicted glycemic index (pGI) of bean-based products. The starch, saccharose and dietary fiber fractions are indicated as g/100 g. <LOQ, under limit of quantification.

Sample		% of Bean Flour in the Total Sample	pGI ^a^	Starch	Saccharose	Dietary Fiber
Cracker 1	wt	26	76.6	46.3	<LOQ	17.9
	*lpa lec* ^−^		71.4	44.6	<LOQ	16.7
	Average		74.0	45.4		17.3
Cracker 2	wt	38	61.6	38.5	<LOQ	22.8
	*lpa lec* ^−^		55.4	36.6	<LOQ	21.7
	Average		58.5	37.5		22.2
Biscuit 1	wt	29 ^b^	42.9	21.2	12.7	14.8
	*lpa lec* ^−^		39.8	19.3	11.3	18.4
	Average		41.3	20.2	12.0	16.6
Biscuit 2	wt	14 ^b^	50.4	18.6	28.5	17.7
	*lpa lec* ^−^		45.0	14.8	32.3	18.2
	Average		47.7	16.7	30.4	17.9

^a^ white bread as control (pGI = 70); ^b^ percentage adjusted on the weight of defatted samples; LOQ = limit of quantification.

**Table 6:** Iron content in wild type (wt) and *lpa lec*^−^ bean flours and Biscuit 1 flours.

Sample	Fe Content (mg/Kg)
wt bean flour	27.03
*lpa lec*^−^ bean flour	29.76
wt Biscuit 1 flour	29.38
*lpa lec*^−^ Biscuit 1 flour	30.13

## Data Availability

Not applicable.
